# Spectroscopic Characterization of 3-Aminoisoxazole, a Prebiotic Precursor of Ribonucleotides

**DOI:** 10.3390/molecules27103278

**Published:** 2022-05-20

**Authors:** Alessio Melli, Mattia Melosso, Kevin G. Lengsfeld, Luca Bizzocchi, Víctor M. Rivilla, Luca Dore, Vincenzo Barone, Jens-Uwe Grabow, Cristina Puzzarini

**Affiliations:** 1 Scuola Normale Superiore, Piazza dei Cavalieri 7, 56126 Pisa, Italy; alessio.melli@sns.it (A.M.); vincenzo.barone@sns.it (V.B.); 2 Dipartimento di Chimica “Giacomo Ciamician”, Università di Bologna, Via F. Selmi 2, 40126 Bologna, Italy; luca.bizzocchi@unibo.it (L.B.); luca.dore@unibo.it (L.D.); 3 Scuola Superiore Meridionale, Università di Napoli Federico II, Largo San Marcellino 10, 80138 Naples, Italy; 4 Institut für Physikalische Chemie und Elektrochemie, Gottfried Wilhelm Leibniz Universität Hannover, Callinstraße 3A, 30167 Hannover, Germany; kevin.lengsfeld@pci.uni-hannover.de (K.G.L.); jens-uwe.grabow@pci.uni-hannover.de (J.-U.G.); 5 Centro de Astrobiología (CSIC-INTA), Ctra. de Ajalvir Km. 4, Torrejón de Ardoz, 28850 Madrid, Spain; vrivilla@cab.inta-csic.es

**Keywords:** rotational spectroscopy, large amplitude motion, astrochemistry, prebiotic molecules, origin of life

## Abstract

The processes and reactions that led to the formation of the first biomolecules on Earth play a key role in the highly debated theme of the origin of life. Whether the first chemical building blocks were generated on Earth (endogenous synthesis) or brought from space (exogenous delivery) is still unanswered. The detection of complex organic molecules in the interstellar medium provides valuable support to the latter hypothesis. To gather more insight, here we provide the astronomers with accurate rotational frequencies to guide the interstellar search of 3-aminoisoxazole, which has been recently envisaged as a key reactive species in the scenario of the so-called RNA-world hypothesis. Relying on an accurate computational characterization, we were able to register and analyze the rotational spectrum of 3-aminoisoxazole in the 6–24 GHz and 80–320 GHz frequency ranges for the first time, exploiting a Fourier-transform microwave spectrometer and a frequency-modulated millimeter/sub-millimeter spectrometer, respectively. Due to the inversion motion of the −NH_2_ group, two states arise, and both of them were characterized, with more than 1300 lines being assigned. Although the fit statistics were affected by an evident Coriolis interaction, we were able to produce accurate line catalogs for astronomical observations of 3-aminoisoxazole.

## 1. Introduction

In the context of the RNA-world hypothesis [1], it is assumed that RNA represents the first self-replicating chemical unit, and thus played a major role in the origin of life on primordial Earth. Although this is a widely accepted hypothesis, it is still debated as to how RNA units could have formed under the physicochemical conditions of early Earth. As it is formally composed of ribose, a phosphate unit, and a nucleobase, it was initially believed that these were the three key ingredients to form RNA. However, the assembling process of these chemical bricks has been shown to be inefficient [2,3], thus leading to the formulation of alternative pathways for the formation of RNA. Laboratory experiments [4] have demonstrated the feasible production of a pyrimidine nucleotide starting from different reactants and under plausible geochemical conditions associated with early Earth. In the model of Powner et al. [4], simple chemical species such as glycolaldehyde, cyanamide, glyceraldehyde, and cyanoacetylene are the required building blocks and 2-aminooxazole constitutes a key intermediate that is first transformed into an arabinose nucleotide and then into the pyrimidine ribonucleotide. Although quite efficient for pyrimidine bases, this process is not able to account for the formation of purine bases. Other hypotheses have been formulated for a prebiotic production of purine nucleotides [5,6], but the required physicochemical conditions are different and not compatible with those proposed by Powner et al. [4]. Recently, a unified and more comprehensive route has been introduced to explain the simultaneous formation of pyrimidine and purine nucleotides [7]. In the scenario proposed by Becker et al. [7], small molecules and ribose are still needed, while the key reactive species is now represented by 3-aminoisoxazole. The process proposed by Becker et al. [7] is extremely efficient because it does not require either any purification steps—all intermediates being formed selectively and robustly—or strict environmental conditions. Thus, it appears to be one of the most valid scenarios investigated to date.

However, a crucial point is how the chemical building blocks essential for initiating such processes were made available on early Earth. According to the exogenous delivery theory [8], prebiotic species could have been transferred to the primitive Earth by meteoritic and cometary bombardment [9,10,11,12,13,14,15]. Therefore, the detection of the so-called interstellar Complex Organic Molecules (COMs, carbon-bearing species containing at least 6 atoms [16]) can be used to support this theory in providing useful hints on the RNA hypothesis. In fact, the simplest species mentioned by both Becker et al. [7] and Powner et al. [4] have been already detected in the interstellar medium (ISM): cyanoacetylene [17], cyanamide [18], hydroxylamine [19], glycolaldehyde [20] and its tautomer ethenediol [21], and urea [22]. Another important piece of information is the very recent observation of ring molecules and polycyclic aromatic hydrocarbons in the ISM [23,24,25,26], which indicates that the chemical complexity found in astronomical objects by far exceeds the expectation and demands searches of larger and more complex COMs, also including aromatic species [27,28].

As mentioned above, 3-aminoisoxazole can play an important role in the elucidation of the RNA-world hypothesis [29,30,31] and its interstellar detection would provide a crucial piece of the origin-of-life puzzle. However, laboratory data are available for its isomer 2-aminooxazole [32], which has been unsuccessfully searched for [33], but not for 3-aminoisoxazole. For this reason, here we report the first observation and analysis of the rotational spectrum of 3-aminoisoxazole, which has been investigated from the microwave (MW) to the millimeter-wave (mmW) regions. Such a result could only be accomplished by a joint theoretical–experimental study.

## 2. Results and Discussion

**Figure 1:**
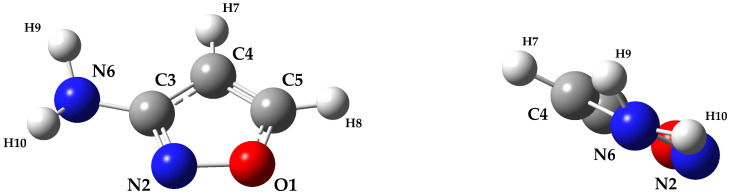
Molecular structure and atom labeling of 3-aminoisoxazole. On the right, a side view of the molecule shows the hydrogen position with respect to the ring plane.

To locate the minimum energy structure of 3-aminoisoxazole, a preliminary scan of its potential energy surface has been carried out at the revDSD level (for its definition, the reader is referred to the Section 3). The investigated internal coordinates, namely the H9-N6-C3-N2 and H10-N6-C3-N2 dihedral angles (for the atoms labeling, see Figure 1), have been varied in order to identify the most stable orientation of the −NH_2_ moiety with respect to the isoxazole ring, with the other structural parameters being optimized at each step. From this analysis, the resulting minimum energy structure is the one shown in Figure 1, which belongs to the symmetry point group $C_{1}$. Indeed, while N6 lays in the plane defined by the atoms of the isoxazole ring, the two hydrogen atoms of the amino group (H9 and H10) are out of this plane (see Figure 1). This orientation is similar to that of several other molecules featuring a planar ring and an amino group, e.g., 2-aminopyridine [34,35], 3- and 4-aminopyridine [36], 2-aminooxazole [32], and aniline [37,38,39,40,41]. For these molecules, the angle between the bisector of the HNH angle and the extension of the C−NH_2_ bond (which is a useful measure of the degree of non-planarity of the −NH_2_ moiety), ranges from 27° to 42°, with the largest value being observed in 2-aminooxazole. For 3-aminoisoxazole, this angle is even larger: 45°. In the rotational spectroscopy studies of the molecules mentioned above, all rotational transitions are split into doublets due to the inversion motion of the −NH_2_ group, and the same effect is expected for 3-aminoisoxazole. This is due to the presence of two equivalent energy minima separated by a planar transition state. For 3-aminoisoxazole, the corresponding double-well potential energy surface has been evaluated at the revDSD level, indeed confirming the presence of two equivalent structures and the planarity of the transition state. To further improve the energetics, the junChS composite scheme (for its definition, the reader is referred to the Section 3) has been employed on top of the revDSD geometries, thus leading to a barrier of 5.1 kJ mol${}^{-1}$ (about 430 cm${}^{-1}$). Comparable values have been found for 2-aminooxazole (5.1 kJ mol${}^{-1}$, see ref. [32]) and aniline (6.4 kJ mol${}^{-1}$, see ref. [41]). For both 3- and 4-aminopyridine, values in the 3–5.4 kJ mol${}^{-1}$ range have also been estimated [36]. Therefore, based on the computed inversion barrier, it is anticipated that the splitting in the rotational transitions should be also observed in 3-aminoisoxazole.

Subsequently, we moved our attention to the computational prediction of the spectroscopic parameters. To guide the experiment, a reliable estimate of the rotational constants is mandatory. To this aim, the revDSD equilibrium structure has been further improved, thereby resorting to the junChS composited scheme. The resulting geometrical parameters are reported in Table 1. As mentioned above, atoms from 1 to 8 lay in the same plane: therefore, the associated dihedral angles have been kept fixed at values of either 0 or 180 degrees. From the junChS geometry, the equilibrium rotational constants have been straightforwardly derived and then augmented by the vibrational corrections evaluated at the revDSD level, thus obtaining the computational estimate of the ground-state rotational constants. Although the accuracy of junChS structures is well documented [42,43], further support from an experiment is desirable. A possible comparison is with the semi-experimental equilibrium structure ($r_{\mathrm{SE}}$) of isoxazole [44]. Based on the assumption that the planar ring is essentially unchanged when moving from isoxazole to 3-aminoisoxazole, the observed average discrepancy of 3 mÅ and 0.1° for bond lengths and angles, respectively, is a confirmation of the accuracy and reliability of junChS equilibrium geometry.

As briefly mentioned in Section 3, the remaining spectroscopic parameters required for the simulation of the rotational spectrum of 3-aminoisoxazole have also been computed (either at the revDSD or junChS level) and are collected in the last column of Table 2.

**Figure 2:**
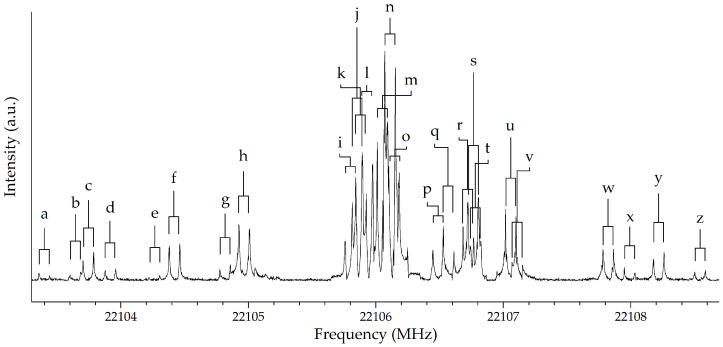
Hyperfine structure of the $J_{K_{a},K_{c}}=3_{1,3}\leftarrow 2_{0,2}$ transition of 3-aminoisoxazole recorded using the COBRA-FTMW spectrometer. The 7 MHz scan has been registered with 10,000 shots every 100 kHz. All lines appear as doublets due to Doppler effect. The labels refer to the spectral lines assigned and collected in the Supplementary Material.

From a spectroscopic point of view, 3-aminoisoxazole is a nearly-prolate asymmetric top (κ=−0.7) with two quadrupolar nuclei, namely N2 and N6. Therefore, its spectrum shows a hyperfine structure due to the coupling between the electric quadrupole moments of the ^14^N atoms ($I_{N}=1$) with the electric field gradient at these nuclei. These interactions lead to the definition of two additional quantum numbers, with the coupling scheme $F_{1}=J+I_{N2}$, $F=F_{1}+I_{N6}$ being adopted. Furthermore, as already discussed above and suggested by similar spectroscopic studies (see, e.g., refs. [32,34,35,36,37,38,39,40,41]), two inversion states are expected because of the large amplitude motion of the −NH_2_ moiety. Relying on the computational prediction of the rotational spectrum, we successfully searched for a few $K_{a}=0,1$ lines using the COBRA Fourier Transform Microwave (FTMW) spectrometer (see Section 3 for instrument details). Their assignment allowed for refined rotational constants and, with an iterative procedure, we completed the analysis of the 6–24 GHz spectrum, thereby assigning more than 200 hyperfine components. Both *a*- and *b*-type transitions have been included. The uniqueness of the hyperfine structure helped in the identifications of the transitions belonging to 3-aminoisoxazole. As an example, the $J_{K_{a},K_{c}}=3_{1,3}\leftarrow 2_{0,2}$ transition is depicted in Figure 2. The experimental resolution of 2 kHz, achievable with the COBRA-FTMW spectrometer, together with its high sensitivity allowed us to assign 28 different components of this line (26 of which are shown in Figure 2). However, in the 6–24 GHz frequency range, no evidence of the second inversion state has been found.

**Figure 3:**
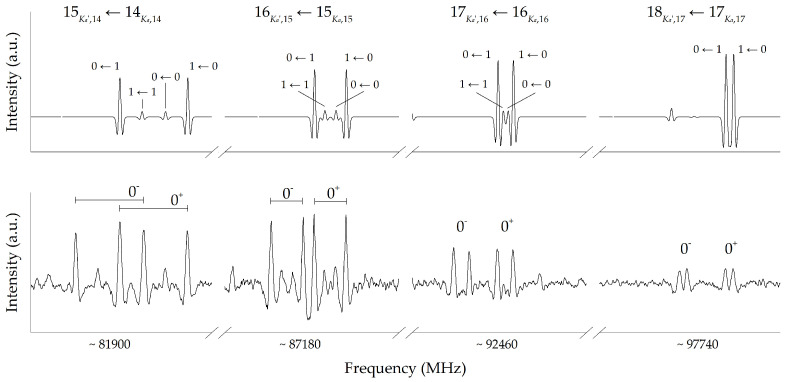
Simulated (based on FTMW results, top panel) and experimental (bottom panel) typical pattern of the $K_{a}=0,1$ transitions in the millimeter-wave region. A 20 MHz scan is shown for each transition. The two inversion states are easily distinguishable.

The measurements in the centimeter-wave region allowed us to determine the ground-state rotational and quartic centrifugal distortion constants, as well as the nuclear quadrupole coupling parameters. These determinations were then used to predict the rotational spectrum of 3-aminoisoxazole in the millimeter-wave region, without, however, accounting for the hyperfine structure, as depicted in the top panel of Figure 3. The choice of neglecting the effects of quadrupole coupling is based on the expectation that, in the millimeter-wave region, the hyperfine splitting is much smaller than the line width. Indeed, the $J_{K_{a},K_{c}}=15_{0,14}\leftarrow 14_{1,14}$ transition, which is the lowest in the frequency range considered in Figure 3, exhibits splitting of about 50 kHz to be compared with a Doppler linewidth (full width at half maximum, FWHM) of 300–400 kHz.

In the 80–320 GHz region, we expected to register a strong *b*-type combined with a less intense *a*-type spectrum, $\mu_{b}$ and $\mu_{a}$ being 3.08 D and 1.19 D, respectively. It has to be noted that the prediction of millimeter-wave transitions based on the FTMW data was affected by negligible errors, especially for the $K_{a}=0,1$ *a*- and *b*-type transitions, on which the impact of the spectroscopic parameter uncertainties was small. Consequently, as shown in Figure 3, the spectral assignment was rather straightforward. From an inspection of the experimental spectra in the bottom panels of Figure 3, the presence of a second set of slightly weaker lines sharing a very similar pattern is immediately apparent. We identified this second group of transitions as belonging to the $0^{-}$ inversion state. After a first estimate of the spectroscopic parameters for the $0^{-}$ state and the inclusion of the measurements in the 80–115 GHz frequency range for the $0^{+}$ state, we were able to extend the characterization up to 320 GHz. No *c*-type transitions were found ($\mu_{c}=1.09$ D), and the reason for this is explained later in the text.

The identification of the $0^{-}$ state in the millimeter-wave spectrum deserves a note at this point. While the centimeter and millimeter/sub-millimeter spectra seem to have contradictory results, the strongly different physical conditions of the two experiments need to be considered. Using the millimeter-/submillimeter-wave spectrometer (FM-mmW), the measurements were carried out at room temperature, with both inversion states thus being populated. Contrary, in the COBRA-FTMW experiment, strong rotational cooling follows the pulsed supersonic expansion in the cavity, thus resulting in a depopulation of the higher-energy $0^{-}$ state. In total, we were able to record and assign more than 1100 and 900 lines for the $0^{+}$ and $0^{-}$ states, respectively, involving energy levels with *J* values up to 68 and $K_{a}$ values up to 25. While the FTMW rotational frequencies were retrieved using the FTMW++ program, PGOPHER [45] was used for the mmW transitions in the 80–320 GHz range.

**Figure 4:**
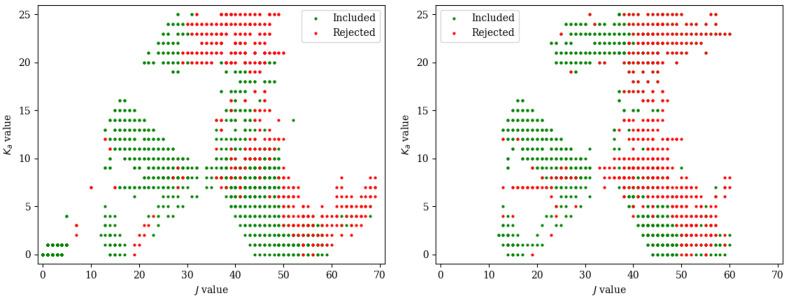
Graphical representation of the included and rejected transitions of the $0^{+}$ (**left** panel) and $0^{-}$ (**right** panel) states in the fitting procedure.

The fit has been performed with the SPFIT subroutines of the CALPGM suite of programs by Pickett [46] and employing a Watson’s S-reduced Hamiltonian [47] in the $I^{r}$ representation. The fitting procedure was rather challenging, and a graphical representation of the difficulties encountered is depicted in Figure 4, which shows the included and rejected lines in terms of the *J* and $K_{a}$ values. All recorded lines could be assigned without any doubt owing to both the accuracy of the spectroscopic parameters and the absence of strong interfering lines in the spectrum. Indeed, the first vibrationally excited state (−NH_2_ wagging) lies at about 266 cm${}^{-1}$ (harmonic value calculated at the revDSD level of theory) and its population at room temperature should be around 28% of that of the ground state. However, while about 1300 lines could be well reproduced in the fitting procedure, more than 600 transition frequencies in the 80–320 GHz range showed an observed–calculated difference more than four times the experimental accuracy (which has been set to 2 kHz for the FTMW experiments and 30 kHz for the FM-mmW ones). All these lines were excluded from the fit after several attempts to incorporate them. On the contrary, no FTMW lines were rejected. These problems are likely due to a Coriolis interaction between the two inversion states. Due to the orientation of the electric dipole components, *a*- and *b*-type transitions occur within each state, while the *c*-type ones connect the $0^{+}$ and $0^{-}$ states. Indeed, the *c* axis is the one perpendicular to the isoxazole ring (see Figure 1), and its sign changes when the inversion motion takes place. Therefore, *c*-type transitions provide a great tool for the determination of the energy difference (ΔE) between the two states, which would be of great help in the description of this interaction. However, the energy involved can be rather high. To further confirm this, we searched for *c*-type transitions in the millimeter-wave range, but we could not find any transition despite the fact that the values of $\mu_{a}$ and $\mu_{c}$ are comparable. For example, for vinylamine, a value of ΔE=45.481(74) cm${}^{-1}$ has been determined [48]; the Coriolis interaction between the inversion states could be described owing to the recording of the $0^{-}\leftarrow 0^{+}$ band [48]. For 2-, 3-, and 4-aminopyrdine, aniline, and 2-aminooxazole, the energy separation was only roughly estimated to be lower than 100 cm${}^{-1}$, thus leading to *c*-type lines taking place in the far infrared region. In the present case, the two states had to be fitted separately with a model that cannot include the energy difference between the two states or the Coriolis interaction between them. As a consequence, as already mentioned, a significant number of transitions could not be incorporated in the analysis. Taking a closer look of Figure 4, the $0^{+}$ state appears to generally behave better with respect to the $0^{-}$ state. Indeed, for the latter, even low $K_{a}$ transitions above J=50 were discarded, together with those with high values of both *J* and $K_{a}$. For both states, no strong deviations were found instead for transitions with *J* below 40 (with the exception of $0^{-}$ lines with $K_{a}=7,8$, which were almost all excluded).

The spectroscopic parameters determined by the fitting procedure described above are collected in Table 2. The agreement between the calculated and experimental parameters is evident. The average relative difference on the rotational constants was found to be about 0.1%, while the discrepancies for quartic centrifugal distortion and nitrogen quadrupole coupling constants are larger, but on average are below 5%. This agreement is in line with the literature on this topic [43,49,50,51,52,53]. Owing to the inclusion in the fit of both *a*- and *b*-type transitions, all rotational constants have been accurately determined: the resulting relative error is about 2 parts per 10 million, while a precision of 2 parts per 10 thousand has been obtained for the quartic centrifugal distortion constants. The inclusion of the sextic centrifugal distortion constants in the fit parameters did not provide any significant improvement to the analysis. Therefore, none of them have been determined and their values have been kept fixed to zero. However, the root mean square (rms) error of the fit reflects the lack in our global Hamiltonian of terms describing the interaction between the two inversion states. Indeed, as the standard deviation of the fit is close to 1.5, the experimental accuracy of the retrieved frequencies has not been well reproduced in the fit. A comparison of our results with respect to those of similar molecules studied in the literature is therefore deserved. For all the species previously mentioned (i.e., 2-aminopyridine [34,35], 3- and 4-aminopyridine [36], 2-aminooxazole [32], and aniline [37,38,39,40,41]), we note that the measurements were carried out below 110 GHz; indeed, the difficulties in our analysis arose above this frequency value. A comprehensive rovibrational work in the far-infrared region on all the species which bear the −NH_2_ moiety together with a planar ring is thus desirable to fill the lack of their spectroscopic characterization. In particular, for 3-aminoisoxazole, this would allow us to overcome the fitting problems related to the neglect of the Coriolis interaction, which led to a not-well-conditioned description of the system.

## 3. Materials and Methods

### 3.1. Computational Details

The investigation of the potential energy surface has been performed at the revDSD-PBEP86-D3(BJ)/jun-cc-pVTZ level of theory (referred in the text to as revDSD), where revDSD-PBEP86 is the latest revision of the double-hybrid DSD-PBEP86 functional [54] and jun-cc-pVTZ is a partially augmented triple-ζ basis set [55,56]. In the level of theory above, D3(BJ) denotes the use of the D3 empirical correction in conjunction with the Becke–Johnson (BJ) damping damping function [57]. To further improve the equilibrium structure determination, the geometry has been optimized using the jun-“cheap” composite scheme (junChS) [58], which is a variant of the so-called “cheap” scheme [59] employing the partially augmented jun-cc-pV*n*Z basis sets [55,56]. The junChS approach is based on the CCSD(T)/jun-cc-pVTZ level (within the frozen-core approximation) improved by the extrapolation to the complete basis limit and the effects of core-valence correlation, both evaluated using the MP2 method [60] (Møller–Plesset perturbation theory to second order). CCSD(T) stands for the coupled-cluster method accounting for single and double excitations and a perturbative treatment of triples [61]. The improvement of the equilibrium structure is crucial for the prediction of spectroscopic parameters because the equilibrium rotational constants are straightforwardly derived from the equilibrium geometry. However, for predicting the rotational spectrum, one needs the vibrational ground-state rotational constants, which can be obtained from the equilibrium ones by adding the vibrational corrections. According to vibrational perturbation theory to the second order (VPT2; see ref. [62] for details), vibrational corrections require the computation of vibration–rotation interaction constants, which in turn involves the evaluation of an anharmonic force field. This has been computed at the revDSD level. To complete the set of molecular and spectroscopic parameters needed for predicting the rotational spectrum, first-order properties, i.e., the electric dipole moment and the nitrogen quadrupole coupling constants, as well as quartic and sextic centrifugal distortion constants, have been calculated at the revDSD level. The nitrogen quadrupole coupling constants were also further improved by exploiting the junChS approach for their evaluation.

### 3.2. Experimental Details

The rotational spectrum of 3-aminoisoxazole has been recorded in the centimeter-wave frequency region (6–24 GHz) using the coaxially oriented beam-resonator arrangement (COBRA) Fourier-transform microwave (FT-MW) spectrometer described in ref. [63]. To expand the sample in the spectrometer cavity, the reservoir of the solenoid valve has been filled with inert glass wool and a few drops of liquid 3-aminoisoxazole (purchased by Sigma Aldrich and used without further purification) have there been deposited. The spectrum has been recorded using a pressure of 1 bar with Ar as gas carrier, usually with five thousand accumulations for strong transitions and up to forty thousand accumulations for the weaker ones. All rotational transitions appear as Doppler doublets due to the coaxial alignment of the resonator with respect to the axis of the molecular jet. For recording the rotational spectrum at higher frequencies (80–320 GHz), a frequency-modulation millimeter-/submillimeter-wave spectrometer (FM-mmW), described in details elsewhere [64,65], has been employed. The radiation source is a Gunn diode (80–115 GHz) coupled with a set of passive frequency multipliers for reaching higher frequencies. The detection system consists of Schottky barrier diodes connected to a lock-in amplifier set at twice the modulation frequency, thus allowing the recording of a 2f spectrum profile. A reservoir of 3-aminoisoxazole was connected to the absorption cell (3.25 m long and 5 cm in diameter); its vapor pressure, together with the vacuum system, was sufficient to generate a flowing pressure of 5 mTorr inside the cell.

## 4. Conclusions

Considered to be a key species in the formation of ribonucleotides by Becker et al. [7], 3-aminoisoxazole is expected to play a role as a crucial prebiotic molecule in the RNA-world hypothesis. Therefore, its first detection in the interstellar medium would provide important insights into the unsolved question of the endogenous or exogenous origin of biomolecules on early Earth. However, to search for 3-aminoisoxazole in interstellar space, its laboratory spectroscopic characterization is a prerequisite. In this work, to asses this requirement, the rotational spectrum 3-aminoisoxazole has been registered in the 6–24 GHz and 80–320 GHz ranges using a Fourier transform microwave spectrometer and a frequency-modulated millimeter/sub-millimeter spectrometer, respectively. To support the experimental investigation, a detailed spectroscopic and energetic characterization was carried out, relying on both density functional theory and composite schemes rooted in coupled-cluster techniques. According to our computational study, a double-well potential is predicted due to the −NH_2_ inversion motion. Indeed, in the millimeter-wave spectrum, two inversion states ($0^{+}$ and $0^{-}$) were observed. More than five hundred transitions (*a*- and *b*-type) could be fitted for each state, thus leading to the determination of two different sets of ground-state rotational and quartic centrifugal distortion constants. Furthermore, the nuclear quadrupole coupling constants due to the presence of two quadrupolar nuclei (the two ^14^N atoms) were obtained for the $0^{+}$ state. We noticed a good agreement between theoretical and experimental spectroscopic parameters. However, a strong Coriolis coupling between the two inversion states prevented experimental accuracy in the fitting procedure: no information on the energy difference between the two states could be derived. Based on the available literature for similar molecules, we assume that the $0^{-}\leftarrow 0^{+}$*c*-type transitions required for the characterization of the Coriolis coupling take place in the far infrared region, thus avoiding any conclusion being drawn in the present work. Despite the difficulties in the analysis, the list of rotational transitions together with the spectroscopic parameters deliver the accuracy needed to guide the astronomical search of 3-aminoisoxazole in the interstellar medium.

## Figures and Tables

**Table 1:** Geometry of 3-aminoisoxazole optimized at the junChS level. Distances in Å and angles in degrees.

Parameter	Value	Parameter	Value
N2-O1	1.3993	C3-N2-O1	105.43
C3-N2	1.3098	C4-C3-N2	111.98
C4-C3	1.4256	C5-O1-N2	108.55
C5-O1	1.3359	N6-C3-N2	121.03
N6-C3	1.3822	H7-C4-C3	128.57
H7-C4	1.0738	H8-C5-O1	115.95
H8-C5	1.0755	H9-N6-C3	113.83
H9-N6	1.0048	H10-N6-C3	112.74
H10-N6	1.0067	H9-N6-C3-N2	143.82
		H10-N6-C3-N2	14.90

**Table 2:** Spectroscopic parameters and fit statistics for 3-aminoisoxazole.

Atom	Parameter	Unit ^1^	Experimental ^2^		Theoretical ^3^
			$0^{+}$	$0^{-}$	
	*A*	MHz	9356.38319(10)	9353.34396(32)	9365.17
	*B*		3669.785470(65)	3667.50297(15)	3673.88
	*C*		2642.302812(47)	2642.25405(11)	2645.93
	$D_{J}$	kHz	0.233858(13)	0.228365(35)	0.22
	$D_{JK}$		1.60119(21)	1.55578(35)	1.49
	$D_{K}$		1.53764(25)	1.57235(33)	1.53
	$d_{1}$		−0.0770640(78)	−0.076715(14)	−0.072
	$d_{2}$		−0.0217051(39)	−0.0240627(65)	−0.022
N2	32χaa	MHz	8.6397(17)	–	8.97
	14(χbb−χcc)		−0.74480(68)	–	−0.79
N6	32χaa		3.8032(25)	–	3.64
	14(χbb−χcc)		1.39391(64)	–	1.46
	$\left(\mathrm{obs}.y/n\right)|\mu_{a}|$	D	(y)	(y)	1.19
	$|\mu_{b}|$		(y)	(y)	3.08
	$|\mu_{c}|$		(n)	(n)	1.09
	#lines	ad.	623	529	
	$J^{\min},J^{\max}$		0, 68	0, 60	
	$K_{a}^{\min},K_{a}^{\max}$		0, 25	0, 24	
	σ		01.47	01.50	
	rms	kHz	40.40	44.80	

^1^ If not specified, the unit is the same as the preceding line one. ^2^ One standard deviation error is reported in parentheses. ^3^ The junChS equilibrium rotational constants have been augmented by the revDSD vibrational contributions. Quartic centrifugal distortion constants and dipole moments components have been determined at the revDSD level of theory, while nuclear quadrupole coupling constants have been calculated using the junChS approach.

## Data Availability

Not applicable.

## Supplementary Materials

The following supporting information can be downloaded at: https://www.mdpi.com/article/10.3390/molecules27103278/s1, machine-readable list of assigned rotational transitions frequencies, spectroscopic parameters, and fit statistics.
